# 

**Published:** 1898-10-08

**Authors:** 


**Notices of Births, Deaths, and Marriages** are inserted in this column at a charge of 2s. 6d. for an announcement not exceeding four lines.

### NOTICE TO CORRESPONDENTS.

All MS., Letters, Books for Review, and other matters intended for the Editor should be addressed The Editor, "The Hospital," The Hospital Building, 28 & 29, Southampton Street, Strand, W.C.

The Editor cannot undertake to return rejected MS., even when accompanied by stamped directed envelope.

**Notice to Advertisers and Subscribers.**

All Advertisements, Orders for Copies of The Hospital, and Business Communications should be addressed to **THE MANAGER (Not to the Editor)**, The Hospital, The Hospital Building, 28 & 29, Southampton Street, Strand, London, W.C.

The Cost of Subscription, post free, is as follows:—Per week, 2½d.; per quarter, 2s. 8d.; per half-year, 5s. 3d.; per annum, 10s. 6d. Foreign:—Per annum, 15s. 2d., payable, in all cases, in advance.

**NOTICE.—Vol. XXIV. of "The Hospital" (April, 1898, to September, 1898), in handsome cloth binding, will shortly be on sale at the Office of this Journal, price 7s. 6d. Cases for binding the Numbers contained in this Volume 1s. 6d. Index to Vol. XXIV., 2½d., post free.**

**The Monthly Part for October is now ready. Price 6d., by post 8d.**

### BOOKS RECEIVED.

Simpkin, Marshall, Hamilton, Kent, and Co.

"Clinical Observations on Two Thousand Obstetric Cases." By G. Porter Mathew, M.D.

Hodder and Stoughton.

"The Unconscious Mind." By Alfred T. Schofield, M.D.

Smith, Elder, and Co.

"Skiagraphic Atlas." By John Poland, F.R.C.S.

J. and A. Churchill.
"Diet and Food." By Alexander Haig, M A., M.D., F.R.C.P.

George Bell and Sons.
"The Wanton Mutilation of Animals." By George Fleming, C.B., LL.D., F.R.C.V.S.

### The Student of Medicine.

### APPOINTMENTS.

R. C. Buist, M.D., appointed Medical Officer in the Department of Obstetrics and Gynæcology of the Dundee Royal Infirmary. L. M. Cairns, M.B., C.M.Edin., appointed House Surgeon to the Cumberland Infirmary, Carlisle. H. J. Crossley, M.R.C.S.Eng., L.R.C.P.Lond., appointed Junior Resident Assistant Medical Officer to the Crumpsall Workhouse Infirmary, Manchester. F. Dommisse, M.B., Ch.B., appointed Clinical Assistant to the Chelsea Hospital for Women.

### THE MEDICAL SCHOOLS.

The following Entrance Scholarships and certificates have been awarded at Guy's Hospital Medical School: Scholarship for University Students—H. S, French, Christ Church, Oxford, £50; R. I. Claxton, King's College, Cambridge, certificate. Open Science Scholarships—E. H. B. Milsom, Guy's Hospital Medical School, £150; F. Rogerson, Guy's Hospital Medical School, and N. I. Spriggs, private study, equal, £30 each; H. D. Smart, Guy's Hospital Medical School, certificate; H. S. Brown, Guy's Hospital Medical School, certificate. Open Arts Scholarships—R. F. MacDonald, Bancroft's School, £100; W. G. Taylor, Aberdeen University, £50; J. W. Walton Folkestone Grammar School, £30; G. Hamilton, Aske's School, New Cross, certificate; G. A. Horton, University Tutorial College, certificate, J. James, St. Bees School, certificate.

At St. Thomas's Hospital Medical School the following entrance scholarships in natural science have been awarded: Charles Michael Roberts Scholarship, £150; Harry Mellor Woodcock Scholarship, £60; Charles Hugh Latham Exhibition, £20.

The following are the results of the examinations for the Entrance Scholarships at the London Hospital Medical College: Price Scholarship, value £120, Mr. F. W. Jones; Epsom Scholarship, value £126, Mr. Colmer; Price University Scholarship, value £60, Mr. Bousfield; Science Scholarship, value £60, Mr. J. W. Fox; Science Scholarship, value £30, Mr. Rainforth.

First and Second Entrance Scholarships at the Middlesex Hospital Medical School have been awarded to Mr. W. Cameron Macaulay and Mr. William Gordon Taylor, M.A., respectively.

The following Entrance Scholarships have been awarded at the Charing Cross Hospital Medical School: Livingstone Scholarship, 100 guineas, to Mr. G. E. Bellamy; Huxley Scholarship, 55 guineas, to Mr. B. R. Bickford; Universities Scholarships, each 60 guineas, to Mr. H. G. Gabb and Mr. B. G. Fiddian. Entrance Scholarships have also been awarded to Mr. R. H. Cooper, 60 guineas; Mr D. M. Davies, 40 guineas; and Mr. T. Law, 30 guineas; and Exhibitions of 30 guineas each to Mr. A. C Ingram, Mr. G. O. Lambert, and Mr. B. R. Lloyd.

### WORKS FOR NURSES.

**A MANUAL FOR HOSPITAL NURSES** AND OTHERS ENGAGED IN ATTENDING ON THE SICK. By EDWARD J. DOMVILLE, L.R.C.P., M.R.C.S. Eighth Edition. Crown 8vo., 2s. 6d.

**A MANUAL OF NURSING, MEDICAL** AND SURGICAL. By C. J. CULLINGWORTH, M.D., F.R.C.P. Third Edition. Fcap. 8vo., 2s. 6d.

**A SHORT MANUAL FOR MONTHLY** NURSES. By C. J. CULLINGWORTH, M.D. Fourth Edition. Revised by M. A. ATKINSON. Fcap. 8vo., 1s. 6d.

**NOTES ON GYNÆCOLOGICAL NURSING.** By J. B. HELLIER, M.D. Fcap. 8vo., 1s. 6d.

**HINTS ON ELEMENTARY PHYSIOLOGY.** By FLORENCE A. HAIG-BROWN. With a Preface by Dr. ORD. With 21 Illustrations. Fcap. 8vo., 1s. 6d.

**LECTURES ON MEDICINE TO NURSES.** By HERBERT E. CUFF, M.D. Second Edition. With 29 Illustrations. Crown 8vo., 3s. 6d.

**ANTISEPTIC PRINCIPLES FOR NURSES.** By C. E. RICHMOND, F.R.C.S. Fcap. 8vo., 1s.

**A SHORT DICTIONARY OF MEDICAL** TERMS. 2s. 6d.

**CHAVASSE'S ADVICE TO A MOTHER** ON THE MANAGEMENT OF HER CHILDREN, and on the Treatment on the Moment of their more Pressing Illnesses and Accidents. Fifteenth Edition by Dr. GEORGE CARPENTER, Physician to the Evelina Hospital for Sick Children. 2s. 6d.

**CHAVASSE'S ADVICE TO A WIFE ON** THE MANAGEMENT OF HER OWN HEALTH, and on the Treatment of Some of the Complaints incidental to Pregnancy, Labour, and Suckling. With an Introductory Chapter especially addressed to a Young Wife. Fourteenth Edition, revised by Dr. FANCOURT BARNES, Consulting Physician to the British Lying-in Hospital. 2s. 6d.

London: J. and A. CHURCHILL,
7, Great Marlborough Street.

### POPULAR TEXT-BOOKS FOR NURSES.

**PRACTICAL POINTS IN NURSING.** For Nurses in Private Practice. By EMILY A. M. STONEY. Crown 8vo., illustrated with 73 Engravings in the Text, and 9 coloured and half-tone Plates. 7/6 net.

"The hints given are excellent, and we hope they may be widely read."—*British Medical Journal.*

**ART OF MASSAGE.** By A. CREIGHTON HALE. Demy 8vo., 200 pp., cloth gilt, profusely illustrated with over 70 special cuts. 6/-

"The latest and most complete book we know on Massage."—*Queen.*

**SURGICAL WARD-WORK AND NURSING.** By ALEXANDER MILES, M.D., Edin.; C.M., F.R.C.S.E. Demy 8vo., cloth boards, illustrated, 3/6.

"The book fills a distinct hiatus in surgical literature."—*Glasgow Medical Journal.*

**"THE BURDETT SERIES" OF POPULAR TEXT-BOOKS ON NURSING.**

Small Crown 8vo. Cloth. 1/- each.

No. 1. **PRACTICAL HINTS ON DISTRICT NURSING.** By AMY HUGHES.

"The whole book bristles with good advice and common sense. It should be in the hands of every district nurse."—*British Medical Journa*

No. 2. **THE MATRON'S COURSE** An Introduction to Hospital and Private Nursing. By Miss S. E. ORME.

"Short practical lectures . . . readable and instructive . . . will prove useful to all who study them."—*Scotsman.*

No. 3. **THE MIDWIVES' POCKET BOOK.** By HONNOR MORTEN, Author of "How to Become a Nurse, and How to Succeed;" &c., &c.

"We have never read a more concise and yet more comprehensive nursing manual."—*Weekly Times and Echo.*

No. 4. **FEVERS AND INFECTIOUS DISEASES:** Their Nursing and Practical Management. By WILLIAM HARDING, M.D.Ed., M.R.C.P. Lond.

"We heartily commend the book to nurses and to mothers of families."—*Aberdeen Free Press.*

No. 5. **NOTES ON PHARMACY AND DISPENSING FOR NURSES.** By C. J. S. THOMPSON.

"The descriptions of the various processes are clear, and the pages are full of useful information. Altogether we are very pleased with the book."—*The Hospital.*

No. 6. **THE RATIONAL USE OF ANTISEPTICS IN MIDWIFERY PRACTICE.** By JAMES MORRISON, M.D., M.B., M.R.C.S., L.R.C.P. Lond. [*In Preparation.*

No. 7. **CARE OF CONSUMPTIVES.** By W. H. DAW, M.R.C.S., L.R.C.P. Lond. [*Just Out.*

London: THE SCIENTIFIC PRESS, Limited, 28 & 29, SOUTHAMPTON STREET, STRAND, W.C.

---

**N.B.—Our Telegraphic Address is "OSPEDALE, London."**

### THE NURSES' BOOKSHELF.

**BURDETT'S OFFICIAL NURSING DIRECTORY, 1898.** Compiled and Edited, with the assistance of a Small Committee of Medical Men and Matrons, by Sir HENRY BURDETT, K.C.B. Containing an outline of the Principal Laws affecting Nurses, particulars of Nurse Training Schools in the United Kingdom and Abroad, Nursing Institutions, &c., and a Directory of Nurses. First Year.—To be Published Annually, crown 8vo., 600 pp., cloth gilt, 5/-

"Useful and well arranged."—*Times*.

"A new work of much value. Nobody compiles books of this kind so well as Sir H. Burdett."—*Athenæum*.

"Cannot fail to be of the greatest service to both the nursing profession and the public."—*Glasgow Herald*.

**THE MOTHER'S HELP AND GUIDE TO THE DOMESTIC MANAGEMENT OF HER CHILDREN.** By P. MURRAY BRAIDWOOD, M.D., formerly Senior Medical Officer to the Wirral Hospital for Sick Children. Second Edition, with Addenda, crown 8vo., cloth 2/-.

"We can confidently recommend the book for the purpose for which it is intended, and it contains many hints that would be valuable to the junior practitioner."—*Bristol Medico-Chirurgical Journal*.

**OUTLINES OF INSANITY.** A Popular Treatise on the Salient Features of Insanity. By FRANCIS H. WALMSLEY, M.D. Demy 8vo., cloth extra, 3/6, Popular edition, stitched, 1/6.

"The work is accurate, and it is well arranged and pleasantly written. It will be serviceable to those for whose use it has been designed."—*British Medical Journal*.

London: THE SCIENTIFIC PRESS, Limited, 28 & 29, SOUTHAMPTON STREET, STRAND, W.C.

N.B.—Our Telegraphic Address is "OSPEDALE, London."

